# Evidence for Enhanced Efficacy of Passive Immunotherapy against Beta-Amyloid in CD33-Negative 5xFAD Mice

**DOI:** 10.3390/biom12030399

**Published:** 2022-03-04

**Authors:** Kathrin Gnoth, Stefanie Geissler, Julia Feldhaus, Nadine Taudte, Victoria Ilse, Sebastian Zürner, Sebastian Greiser, Ulf-Dietrich Braumann, Jens-Ulrich Rahfeld, Holger Cynis, Stephan Schilling

**Affiliations:** 1 Department of Drug Design and Target Validation, Fraunhofer Institute for Cell Therapy and Immunology, Weinbergweg 22, 06120 Halle, Germany; stefanie.geissler@izi.fraunhofer.de (S.G.); julia.feldhaus.99@hotmail.de (J.F.); nadine.taudte@periotrap.com (N.T.); victoria.ilse@izi.fraunhofer.de (V.I.); jens-ulrich.rahfeld@izi.fraunhofer.de (J.-U.R.); stephan.schilling@hs-anhalt.de (S.S.); 2 PerioTrap Pharmaceuticals GmbH, Weinbergweg 22, 06120 Halle, Germany; 3 Department of Diagnostics, Fraunhofer Institute for Cell Therapy and Immunology, Perlickstraße 1, 04103 Leipzig, Germany; sebastian.zuerner@izi.fraunhofer.de (S.Z.); sebastian.greiser@izi.fraunhofer.de (S.G.); ulf-dietrich.braumann@izi.fraunhofer.de (U.-D.B.); 4 Department of Applied Biosciences and Process Technology, Anhalt University of Applied Sciences, Bernburger Straße 55, 06366 Köthen, Germany

**Keywords:** passive immunotherapy, CD33 knock out, amyloid beta, 5xFAD mouse model, Alzheimer’s disease

## Abstract

Passive immunotherapy is a very promising approach for the treatment of Alzheimer’s disease (AD). Among the different antibodies under development, those targeting post-translationally modified Aβ peptides might combine efficient reduction in beta-amyloid accompanied by lower sequestration in peripheral compartments and thus anticipated and reduced treatment-related side effects. In that regard, we recently demonstrated that the antibody-mediated targeting of isoD7-modified Aβ peptides leads to the attenuation of AD-like amyloid pathology in 5xFAD mice. In order to assess novel strategies to enhance the efficacy of passive vaccination approaches, we investigated the role of CD33 for Aβ phagocytosis in transgenic mice treated with an isoD7-Aβ antibody. We crossbred 5xFAD transgenic mice with CD33 knock out (CD33KO) mice and compared the amyloid pathology in the different genotypes of the crossbreds. The knockout of CD33 in 5xFAD mice leads to a significant reduction in Aβ plaques and concomitant rescue of behavioral deficits. Passive immunotherapy of 5xFAD/CD33KO showed a significant increase in plaque-surrounding microglia compared to 5xFAD treated with the antibody. Additionally, we observed a stronger lowering of Aβ plaque load after passive immunotherapy in 5xFAD/CD33KO mice. The data suggest an additive effect of passive immunotherapy and CD33KO in terms of lowering Aβ pathology. Hence, a combination of CD33 antagonists and monoclonal antibodies might represent a strategy to enhance efficacy of passive immunotherapy in AD.

## 1. Introduction

AD is a progressive and incurable neurodegenerative disorder, occurring in mid or late life. To date, 40 million people are affected, making AD the most common neurodegenerative disease worldwide [1]. Despite significant endeavors in drug development, only symptomatic and transiently active therapies are currently available, making AD one of the largest unmet medical needs. In AD patients, two histological alterations are observed post mortem: neurofibrillary tangles, which consist of aggregated forms of hyperphosphorylated protein tau [2] and amyloid plaques. The latter are extracellular deposits, which are primarily composed of Aβ peptides. Aβ is generated by endoproteolytic cleavage of the transmembrane amyloid precursor protein (APP) [3]. According to the widely accepted amyloid hypothesis, the oligomerization and deposition of Aβ initiate a cascade of downstream pathological events including intracellular formation of neurofibrillary tangles, neuronal atrophy and neuroinflammation [4]. Most prominent support for this hypothesis is provided by inherited forms of AD (familial AD, FAD), which are caused by mutations in genes involved in formation or deposition of Aβ.

Therefore, immunotherapeutic approaches targeting Aβ are among the most intensively investigated treatment approaches in recent decades [5,6]). In June 2021, the FDA granted accelerated approval of Aducanumab/Aduhelm, a monoclonal antibody recognizing soluble Aβ oligomers and insoluble fibrils that clears Aβ plaques from the brain in mild cognitive impairment (MCI) and mild AD patients [7]. However, Aducanumab still shows side effects and failed to achieve the desired impact on cognitive decline [8]. The observed side effects in passive immunotherapy are presumably caused by binding to vascular Aβ, thereby causing vasogenic edema and cerebral microhemorrhages [9]. Additionally, there are elevated levels of soluble Aβ in the blood of AD patients, leading to the saturation of anti-Aβ antibodies in the periphery and consequently lowering the effective antibody concentration [5]. One approach to circumvent these shortcomings is targeting modified forms of Aβ. Because these forms are mainly restricted to the brain, the antibody is not captured by circulating Aβ peptides in the periphery. This leads to higher antibody amounts available for crossing the blood–brain barrier (BBB). Antibodies binding post-translational variants of Aβ already come to the fore [10,11,12,13]. Donanemab is the first antibody in clinical development, which is directed to a post-translationally modified form of Aβ (pE3-Aβ) [11]. This antibody recently showed significant removal of amyloid load and cognitive stabilization in a Phase 2 clinical trial called Trailblazer-Alz [14,15]. In an upcoming Phase 3 study (Trailblazer-Alz 4), Donanemab will be compared to Aducanumab concerning amyloid plaque clearance in participants with early symptomatic AD. Eli Lilly plans to finish submitting Donanemab for FDA approval in the next few months, paving the way for a decision in the second half of 2022. Targeting post-translationally modified Aβ may lead to the desired cognitive stabilization, but still goes along with amyloid-related abnormalities (ARIA) [14], providing room for an improvement of passive immunotherapy by combinational therapies, which allows the application of lower doses while increasing the beneficial effects and reducing the adverse effects. 

As a result of genome-wide association studies (GWAS), a number of genes have been linked to the onset and development of sporadic AD, the most prevalent form of the disease. Interestingly, most of these genes are involved in microglial biology, among those the myeloid cell receptor CD33. CD33 or sialic-acid-binding immunoglobulin-like lectin 3 (Siglec-3) is a 67 kDa type 1 transmembrane protein and plays a role in mediating cell–cell interactions and in maintaining immune cells in a resting state. In brain, it is exclusively expressed by microglia and infiltrating macrophages. One of the AD-associated single-nucleotide polymorphisms (SNPs), rs3865444 [16,17], is located within the proximal promoter of CD33. The more common allele rs3865444C (>70%) has been found to be associated with an increased risk of AD [18,19,20]. On the other hand, the minor allele rs3865444A, which is associated with a robust increase in the proportion of alternative splicing of non-functional CD33, confers protection against AD. The protective effect is presumably mediated by monocytes/microglia, which showed an enhanced phagocytosis of amyloid plaques in vitro [21]. Furthermore, an elevated expression of CD33 could be shown on the surface of microglia from AD patients. A correlation of CD33-positive microglia with the amount of amyloid plaques in AD brain was observed [22]. Griciuc and colleagues generated an APP(Swe)/PS1(ΔE9)/CD33KO mouse line and found that knocking out CD33 results in lower Aβ levels and reduced amyloid plaque burden in the brain. Since these mice do not exhibit altered amyloid precursor protein processing, this phenotype is likely associated with Aβ clearance. 

Hence, CD33 represents an important regulator in the process of phagocytosis. Because antibody-mediated phagocytosis of Aβ by microglia is supposed to be the main mode of action in anti-Aβ immunotherapies, we wanted to address the question, how CD33KO affects the efficacy of passive immunotherapy. In order to test this hypothesis, we generated a new mouse line by generating heterozygous 5xFAD mice in the background of a homozygous CD33KO. These mice were subjected to treatment with a previously described monoclonal antibody (K11), which targets the post-translational modified peptide isoaspartate 7-Aβ (isoD7-Aβ) [12]. 

The results were intended to provide implications, whether a CD33KO shows an additive effect on the Aβ-lowering capacity of passive vaccination. 

## 2. Materials and Methods

### 2.1. Antibodies

Purified monoclonal 4G8 and 4G8-HRP were obtained from Biolegend, San Diego. For application to 5xFAD and 5xFAD/CD33KO mice, K11 was recombinantly expressed with an IgG2a subtype in Freestyle 293-F cells (Thermo Fisher Scientific, Braunschweig, Germany) by using the bicistronic vector pVITRO1-neo-mcs (InvivoGen). The isotype control antibody originally possesses an IgG2a subtype and was expressed in hybridoma cells. Antibody purifications from hybridoma or Freestyle 293-F supernatants have been performed by Protein G affinity chromatography. Bound antibodies were eluted using 100 mM Glycin-HCl, pH 2.7 and dialyzed twice against D-PBS overnight at 4 °C.

### 2.2. Animal Treatment Studies

#### 2.2.1. Ethical Statement

All experimental procedures were performed in compliance with animal welfare policies and approved by the local authorities (Landesverwaltungsamt Sachsen Anhalt, Referat 203 Verbraucherschutz, Veterinärangelegenheiten, approval number 42502-2-1499). The personnel involved in the experiments hold the required certificates to perform animal experiments. Mice are housed under specific pathogen-free (SPF) conditions and are monitored quarterly concerning infections with specific pathogens according to the FELASA recommendations [23]. 

#### 2.2.2. Experimental Animals and Housing

CD33KO and 5xFAD mice are both bred on a C57BL/6J background and were obtained from The Jackson Laboratory (Cd33tm1Avrk, Order #006942 [24] and Tg (APPSwFlLon, PSEN1*M146L*L286V) 6799Vas, MMRRC stock #34840 [25]. Since we performed our treatment studies with heterozygous 5xFAD mice carrying a homozygous CD33KO (termed 5xFAD/CD33KO in the following text), we mated heterozygous 5xFAD/heterozygous CD33KO animals with homozygous CD33KO mice. The genotype of every descendant was determined by specific PCR protocols. In detail, at weaning age of 19–21 days, ear punches were taken to individually mark the mice. The left-over ear tissue was used for genotyping. DNA was isolated from the ear punches using the 1-Step kit (Nexttec, Leverkusen, Germany) according to the manufacturer’s protocol. The DNA was analyzed for the presence of the 5xFAD transgene and CD33KO allele using the GoTaq DNA Polymerase (Promega, Madison, WI, USA). Primers were for 5xFAD: Forward: 5′- CTA GGC CAC AGA ATT GAA AGA TCT-3′, Reverse: GTA GGT GGA AAT TCT AGC ATC ATC C-3′. The 5xFAD PCR protocol does not discriminate between heterozygous and homozygous genotype. Therefore, 5xFAD were mated on a heterozygous background using WT C57BL/6J mice. Genotyping of CD33KO applied the following primers: Common Forward: 5′-GCT TCT GCC ACA TAC TCA TTC A-3′, WT Reverse: 5′-CAA GGG GTT AAC AGA GGA ACC-3′ and CD33KO, Reverse: 5′-CCA GAG GCC ACT TGT GTA GC-3′. The PCR protocol for CD33KO allowed the discrimination of WT (231 bp) and KO (172 bp) alleles. Samples were loaded onto a 1.6% agarose gel in TAE buffer with ethidium bromide to visualize the PCR products. A Severity Assessment required by German law for newly generated and genetically-modified mouse lines (here: 5xFAD/CD33KO) was performed according to the regulations of the ‘Bundesinstitut für Risikobewertung’ (BfR). As a result of this analysis, we found no abnormalities or harm burdens different than for 5xFAD mice or CD33 KO mice alone. The average litter size of 5xFAD/CD33KO was found to be in the normal range with 7.8 ± 2.3 pups per female. Furthermore, observation of 117 newborn pubs until weaning revealed 3 losses until weaning (2.5%) and was also considered normal.

#### 2.2.3. Study Design

In order to define the minimal treatment dose, 3-month-old female heterozygous 5xFAD mice (9 animals per group) were treated intraperitoneally with 12 mg/kg (~300 µg/mouse), 4 mg/kg and 1.3 mg/kg K11 once a week in an initial treatment trial. As a negative control (anti-Hanta Virus IgG2a), isotype control antibody was applied in a dose of 12 mg/kg once a week. Mice were sacrificed after 24 weeks of treatment. 

In a second treatment trial, 3-month-old female heterozygous 5xFAD and 5xFAD/CD33KO mice were treated with 4 mg/kg K11 and IgG2a isotype control. The group size was 8–10 animals per group. After 38 weeks of treatment, an Elevated Plus Maze (EPM) test was performed, followed by the preparation of mouse brain and ELISA analysis for Aβ quantification or immunohistochemical analyses.

Animals were randomly assigned to the treatment groups. In order to authenticate and reduce risk of mixing up heterozygous 5xFAD and 5xFAD/CD33KO animals, genotyping of every animal was conducted at start and at the end of study. Executive staff involved in immunization, EPM tests, immunohistochemical and ELISA analysis were blinded. Animals were labeled by ear tags, permitting no conclusions to group assignment.

#### 2.2.4. Sample Collection

Mice were sacrificed using CO_2_ inhalation one week after the final immunization. Plasma collection, PBS perfusion, brain removal and treatment of both hemispheres was already described [12].

#### 2.2.5. Preparation of T-Per and 5 M Guanidine Hydrochloride (5 M GdmCl) Brain Fractions

In order to prepare mouse brain for isoD7-Aβ and total Aβ-ELISA analysis, the left hemisphere was homogenized in T-Per buffer (Tissue Protein Extraction Reagent, Thermo Fisher Scientific) at a concentration of 50 mg/mL with Protease Inhibitor Cocktail Tablets (Roche) by using a Precellys homogenizer (VWR), followed by sonification for 10 s. The homogenate was centrifuged for 1 h at 100,000× *g*, thereby yielding the T-Per fractions. The resulting pellet was dissolved to 150 mg/mL in 5 M GdmCl, followed by an incubation step in an overhead shaker for 3 h at room temperature. After a next centrifugation step (1 h at 100,000× *g*), supernatant (5 M GdmCl fractions) was collected and stored at −20 °C until use.

#### 2.2.6. Elevated plus Maze (EPM) Test

Conduction of this test was already described [12]. In short, test animals were placed with their head to the end of a defined closed arm of an elevated, plus-shaped (+) maze with two open and two enclosed arms (Biobserve GmbH, Bonn, Germany). Subsequently, spontaneous exploration of the maze was recorded for 10 min. The time the animals spent in the open arms was summed up in order to calculate the percentage of time spent in exposed area. 

### 2.3. Immunohistochemical Readout

#### 2.3.1. Tissue Preparation and Immunohistochemistry

Mice were sacrificed by CO_2_ asphyxiation, brains were removed, post-fixed in 4% buffered paraformaldehyde and cryoprotected in 30% sucrose in 0.1 M phosphate buffer (pH 7.4). Sagittal sections from hippocampal region (30 µm) were cut on a cryomicrotome (Cryostar NX70) and collected in 0.1 M phosphate buffer containing 0.025% sodium azide. Brain sections were pre-treated with 60% methanol, 1% H_2_O_2_, followed by washes in 0.1 M TBS (pH 7.4) and blocked in TBS containing 0.3% TritonX-100 and 5% normal goat serum to reduce unspecific binding of antibodies. For staining of total Aβ, brain sections were incubated with 2 µg/mL of commercially available antibody 3A1 (Biolegend GmbH, Germany). For staining of isoD7-Aβ 2 µg/mL of the antibody K16 (generated at Fraunhofer IZI-MWT [12]) were used. Then, sections were incubated with biotinylated anti-mouse antibody (1:1000 Dianova, Hamburg) in 0.1 M TBS with 2% (*v*/*v*) BSA, followed by incubation with ExtrAvidin-peroxidase (Sigma-Aldrich, diluted 1:1000 in 2% [*w*/*v*] BSA in 0.1 M TBS). After washing steps, immunostaining was performed by treatment of sections with 0.05% (*w*/*v*) of the chromogenic substrate 3,3′-diaminobenzidin (DAB) in 0.05 M Tris, pH 7.6 with 0.015% (*v*/*v*) H_2_O_2_.

#### 2.3.2. Quantification of Immunohistochemical Labeling

All pictures were recorded by using the microscope Biorevo BZ-9000 (Keyence, Neu-Isenburg, Germany) with transmitted light modus and an exposure time of 1/200 s. The proportion of isoD7-Aβ- and total-Aβ-labelled structures (region of interest [ROI] in%) was quantified based on overall area of ROI by using the program BZ II Analyzer.

#### 2.3.3. Immunohistochemical Quantification of Plaque-Surrounding Microglia

The nuclei of microglia were stained using the antibody PU.1 by using the DAB staining protocol. The same sections were additionally subjected to Congo Red staining, applying the protocol of Wilcock et al., 2006 [26]. Pictures were recorded with the slide-scanner-microscope Axio Scan Z.1 (Zeiss). Plaque-adjacent microglia have been counted within a predefined region in cortex and hippocampus. In these regions, ten randomly chosen amyloid plaques were analyzed. Each amyloid plaque-surrounding was analyzed with the program ZEN 3.3 (blue edition) depending on the plaque size. In the defined areas, the microglia nuclei were manually counted. For quantification, a mean was calculated based on the numbers of microglia surrounding ten amyloid plaques in each region (in cortex and hippocampus) per brain section.

#### 2.3.4. Determination of Median Aβ Plaque Size and Aβ Plaque Counts

For determination of median Aβ plaque size and Aβ plaque counts, brain sections stained for total Aβ by using antibody 3A1 have been used. Forty entire brain sections were imaged by the use of a slide scanner Zeiss Axio Scan.Z1 with a 20x/0.8 NA objective leading to images with a size of approximately one gigapixel and four fluorescent channels each. The images were processed by a self-developed macro for Fiji [27]. In a preprocessing step, the channels were normalized and enhanced in contrast as well as cleaned from artifacts from the stitching procedure of the slide scanner or uneven illumination. Afterwards, the region of interest was manually separated. Prior to determination of median Aβ plaque size and Aβ plaque counts, a median filter was applied to account for noise. To further address uneven illumination and inhomogeneous staining, the images were binarized with a local mean thresholding algorithm using the same set of parameters for every single image. A binary opening followed by a binary closing clears out very small plaques, fills holes and smooths plaque contours. Plaques with a diameter below 10 µm were neglected. In the last step, information is being extracted regarding the plaque size and the count of plaques for each image with the Fiji tool *analyze particles*.

### 2.4. ELISA Readout

The implementation of the isoD7-Aβ ELISA (using K11 for capturing) and total Aβ-ELISA (using antibody 3D6 for capturing) analysis was performed as described in Gnoth et al., 2020 [12]. 

## 3. Results

### 3.1. Identification of Minimal Antibody Treatment Dose—Pilot Study

With regard to the results observed in the APPSwe/PS1ΔE9/CD33−/− mice [22], a less pronounced AD phenotype was also predicted in the newly generated 5xFAD/CD33KO mouse line. As a result of antibody treatment, we expected a therapeutic add-on effect of 5xFAD/CD33KO mice in comparison to 5xFAD mice. Since 5xFAD mice potentially show an advanced AD phenotype anyway, it might have been difficult to demonstrate the therapeutic add-on effect when using the maximum antibody dosage. Additionally, the use of adequate drug combinations should allow the application of lower doses of each constituent. Hence, we initially performed a dose-finding study with K11 in 5xFAD mice. Therefore, we applied different doses (12, 4 and 1.3 mg/kg) of K11 in comparison to 12 mg/kg isotype control antibody weekly for 24 weeks to 3-month-old 5xFAD mice (Figure S1). A 24-week treatment with 1.3 mg/kg K11 has no effect on insoluble brain Aβ level, while treatment with 4 mg/kg shows a visible reduction. Treatment with 12 mg/kg K11 resulted in a significant decrease in isoD7-Aβ load (*p* = 0.0474). With regard to the findings of our pilot study, we decided to use the minimal efficient dosage of 4 mg/kg for further analyses. This antibody concentration just showed a visible effect, without reaching significance. The usage of the resulting minimal active antibody treatment dose should visualize the anticipated add-on effect of CD33 knock out, even with regard to an apparent low difference of antibody effects in 5xFAD compared to 5xFAD/CD33KO mice. It was already known from earlier 38 week treatment studies that using 12 mg/kg K11 resulted in a significant reduction in brain Aβ level [12].

### 3.2. Treatment of 5xFAD and 5xFAD/CD33KO Mice with K11—Treatment Study

In a second treatment trial, 3-month-old 5xFAD and 5xFAD/CD33KO mice were subjected to 36 weeks of weekly intraperitoneal injections of 4 mg/kg K11. Negative control groups of 5xFAD and 5xFAD/CD33KO mice received weekly injections of 4 mg/kg IgG2a isotype antibody. In the course of the treatment period, mice were subjected to an EPM test at 9 months of age and at 12 months of age, before final sample collection for ELISA and immunohistochemical readouts.

#### 3.2.1. CD33KO Decreases Amyloid Plaque Load in 5xFAD Mice and Leads to an Add-On Effect in Passive Immunotherapy

**Figure 1:**
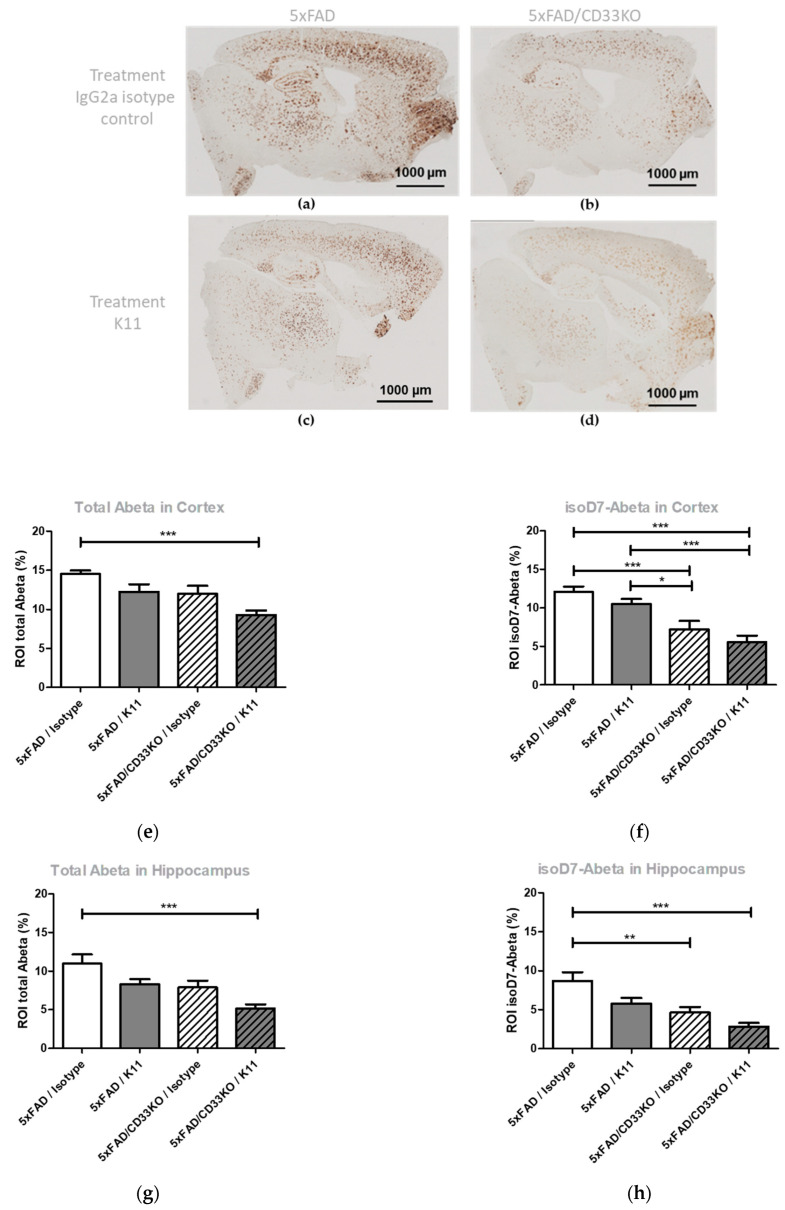
Immunohistochemical analysis of Aβ aggregates in brain sections from 5xFAD and 5xFAD/CD33KO mice treated with K11 or isotype control. (**a**–**d**) Immunohistochemical analysis. Representative images of 30 µM sections of the right brain hemisphere from 12-month-old 5xFAD and 5xFAD/CD33KO. Treatment was 4 mg/kg K11 or isotype control once weekly. ROI in brain sections were selected by staining with anti-isoD7-Aβ antibody K16 or total Aβ antibody 3A1 as indicated, followed by application of biotinylated anti-mouse IgG1. (**e**–**h**) Quantitative evaluation. Area of isoD7- or total Aβ-containing peptides (ROI in%) was quantified based on overall area of ROI by using the program BZ II Analyzer. For statistical analysis, Tukey’s Multiple Comparison Test was used. Sample size was at least 8 animals, with a maximum of 10 animals per group. * means *p* ≤ 0.05; ** means *p* ≤ 0.01; *** means *p* ≤ 0.001. The error bars represent the SEM.

For visualization of Aβ plaque load, we performed immunohistochemical staining of Aβ plaques by using the antibody 3A1, which binds the N-terminus of non-modified total Aβ [28] and by using an anti-isoD7-Aβ antibody K16 [12]. Figure 1 shows the detected amyloid plaque load in cortex and hippocampus of the different treatment groups. As already demonstrated in the pilot study, treatment of 5xFAD mice with the minimal treatment dose resulted in a non-significant reduction in amyloid plaque load in cortex and hippocampus (compare Figure 1a,c) as well as 5xFAD/Isotype and 5xFAD/K11 in Figure 1e–h). Interestingly, CD33 knock out not only causes a reduction in total Aβ, the amount of isoD7-Aβ is affected even more (compare the groups 5xFAD/Isotype and 5xFAD/CD33KO/Isotype in Figure 1e–f). A combinational therapy, comprising K11 antibody treatment and CD33KO, leads to the lowest Aβ plaque load and therefore to the highest treatment effect. 

**Figure 2:**
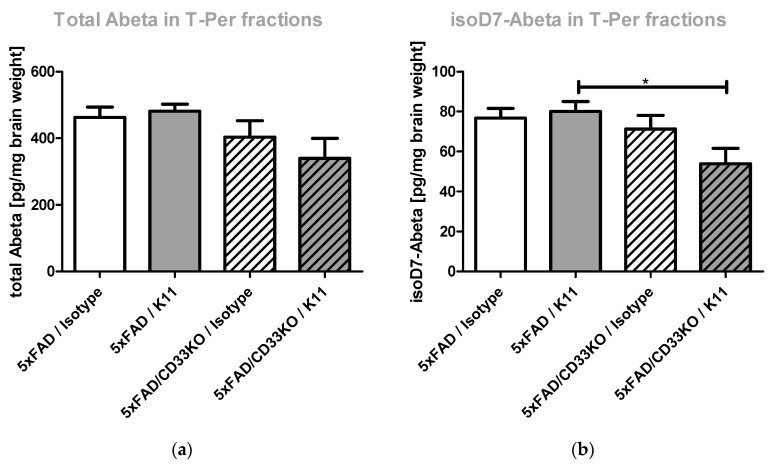

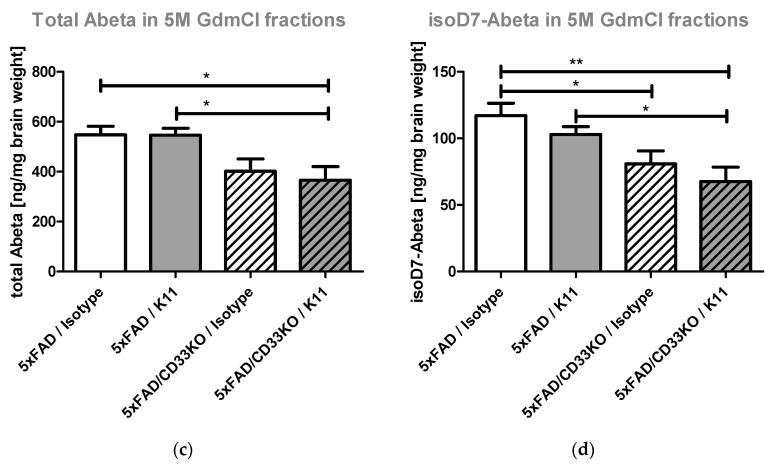
Quantification of total Aβ and isoD7-Aβ peptides in T-Per soluble and 5 M GdmCl soluble brain fractions. Three-month-old 5xFAD and 5xFAD/CD33KO mice were treated intraperitoneally once a week with 4 mg/kg K11 or 4 mg/kg isotype control. After 36 weeks of treatment, mice were sacrificed. The left hemisphere was homogenized in T-Per buffer, followed by centrifugation. The resulting supernatants were applied to a total Aβ (**a**) and isoD7-Aβ (**b**) specific ELISA. The pellet was resuspended in 5 M GdmCl, again centrifuged and the supernatants applied to a total Aβ (**c**) and isoD7-Aβ (**d**) specific ELISA. Sample size was at least 8 animals, with a maximum of 10 animals per group. * means *p* ≤ 0.05; ** means *p* ≤ 0.01. The error bars represent the SEM.

The left hemispheres of the treated mice were used for quantification of isoD7-Aβ and total Aβ brain level in soluble T-Per and insoluble 5 M GdmCl fractions. T-Per contains a mild detergent and was shown to extract target proteins from various cellular compartments, for example from plasma membranes. Mainly monomeric and oligomeric Aβ peptides are supposed to be present in the T-Per fraction. GdmCl is a strong denaturant of folded protein structures, mainly Aβ peptides possessing fibrillary structures are predicted to be dissolved in 5 M GdmCl. ELISA analysis of soluble T-Per fractions showed no effect of minimal dose K11 treatment in 5xFAD genotypes. Interestingly, a reduction in total Aβ and isoD7-Aβ was observed by K11 treatment, in particular from 71 pg/mg isoD7-Aβ in the isotype-treated 5xFAD/CD33KO group to 53.9 pg/mg in the 5xFAD/CD33KO group treated with K11 (Figure 2b). This result clearly shows the add-on effect of CD33KO in passive immunotherapy. ELISA analysis of 5 M GdmCl brain tissue fractions of 5xFAD mice treated with the minimal dose of anti-isoD7-Aβ antibody K11 showed no treatment effect in 5xFAD genotypes concerning total Aβ levels (Figure 2c). However, similar to the results obtained with the T-Per soluble brain fractions, a slight reduction in total Aβ was observed by K11 treatment of 5xFAD/CD33 genotypes (Figure 2c). Considering isoD7-Aβ levels, a significant (*p* = 0.0215) reduction of approximately 31% was observed by CD33KO alone (from 117 ng/mg in the 5xFAD/Isotype group to 81 ng/mg in the 5xFAD/CD33KO/Isotype group, Figure 2d). In combination with K11 treatment, isoD7-Aβ levels have been reduced approximately 42% (from 117 ng/mg in the 5xFAD/Isotype group to 68 ng/mg in the 5xFAD/CD33KO/K11 group). Similar to the results obtained with the soluble T-Per fractions, the amount of isoD7-Aβ is again stronger affected by CD33KO than the total Aβ level.

#### 3.2.2. CD33KO Ameliorates Behavioral Deficits in 5xFAD Mice

The relationship between amyloid burden and cognitive symptoms remains unclear. It was already demonstrated that knocking out CD33 results in lower Aβ levels and reduced amyloid plaque burden in the brain of APP(Swe)/PS1(ΔE9)/CD33KO mice [22]. By conducting an EPM test, the influence of CD33-mediated enhanced plaque clearance on memory deficits was analyzed. The EPM test is a test for the measurement of anxiety and exploratory behavior. Since there is no learning effect on test animals, this test can be performed repeatedly. During the therapy, mice were subjected to an EPM test twice: with 9 months of age after 24 weeks treatment and with 12 months of age after 36 weeks treatment. In 9-month-old animals, we determined neither differences between 5xFAD and 5xFAD/CD33KO groups nor between K11 or isotype control treatment (Figure S2a). At the age of 12 months, treatment of 5xFAD with 4 mg/kg K11 reduced the time animals spent in open arms by 1.6% (from 42.7% in 5xFAD treated with isotype control to 41.1% in K11 treated 5xFAD mice, Figure S2b). Treatment of the 5xFAD/CD33KO mouse line resulted in a reduction of 2.6% in open arms. Importantly, CD33 knock out alone resulted in a decrease of 11.1% in open arms (42.7% in isotype treated 5xFAD versus 31.6% in isotype treated 5xFAD/CD33KO). After conducting Tukey’s Multiple Comparison Test for statistical analysis, no significant differences were found between the different treatment groups. 

#### 3.2.3. CD33KO Increases Number of Plaque-Surrounding Microglia under K11 Treatment

**Figure 3:**
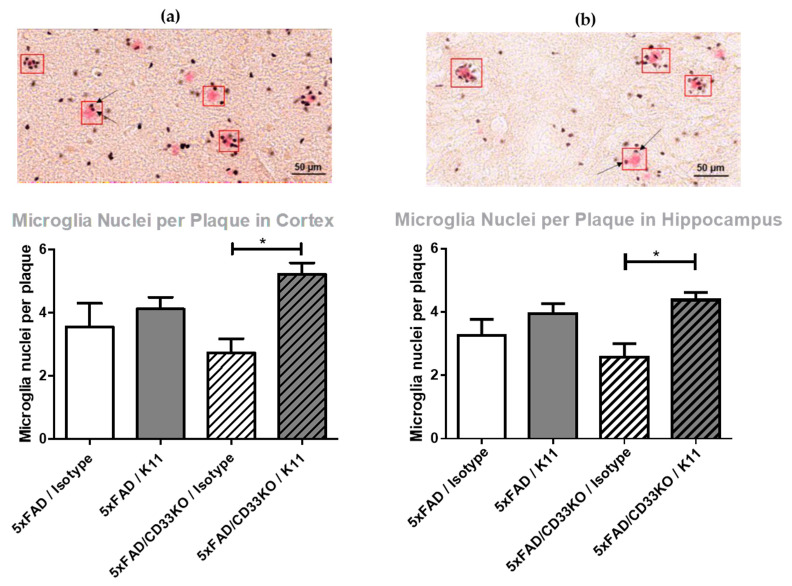
Immunohistochemical quantification of plaque-surrounding microglia in (**a**) cortical and (**b**) hippocampal brain sections from 5xFAD and 5xFAD/CD33KO mice treated with K11 or isotype control. Three-month-old 5xFAD and 5xFAD/CD33KO mice were treated intraperitoneally once a week with 4 mg/kg K11 or isotype control. After 36 weeks of treatment, mice were sacrificed. The right hemisphere was treated with paraformaldehyde, cryopreserved and sectioned. Amyloid plaques were stained by Congo Red in A—cortex and B—hippocampus, and microglia nuclei were stained by antibody PU.1. Plaque-adjacent microglia have been counted within a predefined area (red rectangle) around ten plaques per brain section. For statistical analysis, Tukey’s Multiple Comparison Test was used. * means *p* ≤ 0.05. The error bars represent the SEM.

Since the proposed underlying mechanism of CD33KO-mediated reduction in Aβ plaque load is microglial phagocytosis, immunohistochemical staining of plaque-surrounding microglia has been performed. In order to count plaque-surrounding microglia, amyloid plaques were stained by Congo Red and microglia nuclei by antibody PU.1. Plaque-adjacent microglia have been counted within a predefined area around 10 plaques per brain section, which is exemplarily marked by a red rectangle in Figure 3. We could demonstrate that antibody treatment alone slightly increases plaque-surrounding microglia (from 3.27 microglia nuclei per plaque in the hippocampus of 5xFAD/Isotype group to 3.95 microglia nuclei per plaque in the hippocampus of 5xFAD/K11 group, Figure 3). Surprisingly, CD33KO alone slightly decreases the number of plaque-surrounding microglia (from 3.27 microglia nuclei per plaque in the hippocampus of 5xFAD/Isotype to 2.56 microglia nuclei per plaque in the hippocampus of 5xFAD/CD33KO/Isotype). However, the combination of antibody treatment and CD33KO increases the amount of plaque-surrounding microglia significantly (from 3.27 microglia nuclei per plaque in the hippocampus of 5xFAD/Isotype to 4.38 microglia nuclei per plaque in the hippocampus of 5xFAD/CD33KO/K11, *p* = 0.022).

#### 3.2.4. CD33KO as Well as Antibody Treatment Lead to a Reduction in Amyloid Plaque Counts Rather Than Amyloid Plaque Size 

**Figure 4:**
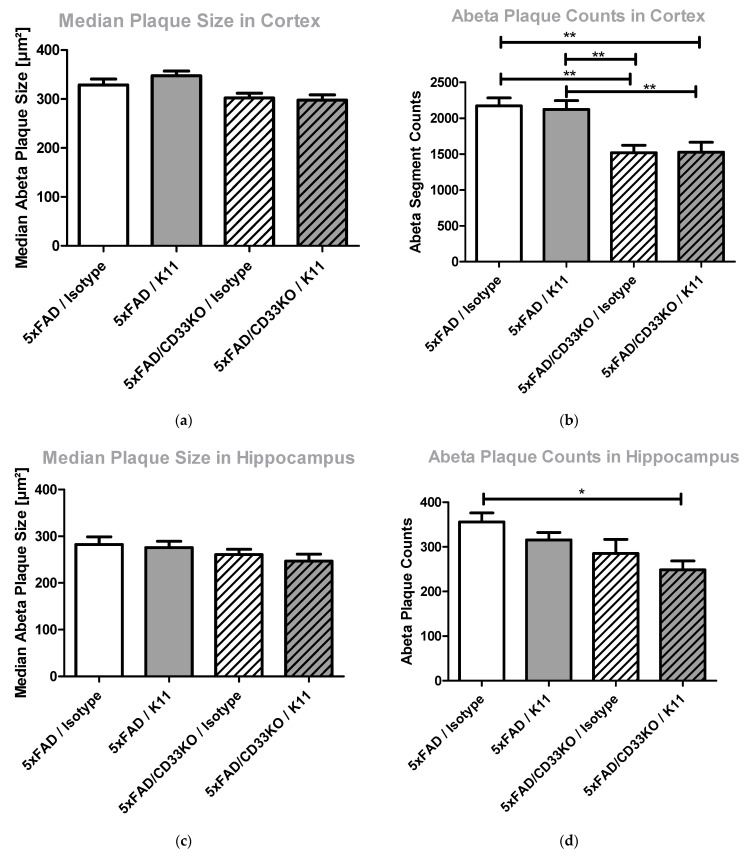
Determination of (**a**) median Aβ plaque size in cortex, (**b**) Aβ plaque counts in cortex, (**c**) median Aβ plaque size in hippocampus and (**d**) Aβ plaque counts in hippocampus in brain sections from 5xFAD and 5xFAD/CD33KO mice treated with K11 or isotype control. Three-month-old 5xFAD and 5xFAD/CD33KO mice were treated intraperitoneally once a week with 4 mg/kg K11 or 4 mg/kg isotype control. After 36 weeks of treatment, mice were sacrificed. The right hemisphere was treated with paraformaldehyde, cryopreserved, sectioned and total Aβ stained by using antibody 3A1. Information regarding plaque size and count of plaques for each image was obtained by using the Fiji tool *analyze particles*. Sample size was at least 8 animals, with a maximum of 10 animals per group. * means *p* ≤ 0.05; ** means *p* ≤ 0.01. The error bars represent the SEM.

Since CD33 is an inhibitory molecule on the surface of brain microglia, it is supposed to impede microglial phagocytosis of amyloid plaques. In order to obtain detailed insight into this mode of action, we used brain sections stained for total Aβ to evaluate the size of Aβ plaques versus Aβ plaque counts. Figure 4 shows the results obtained after analyzing hippocampal and cortical brain sections from 5xFAD and 5xFAD/CD33KO animals treated with K11 or isotype control antibody. We could demonstrate that CD33KO as well as K11 treatment barely reduced the size of amyloid plaques, but the amount of amyloid plaques is significantly (*p* = 0.0174) reduced in the hippocampus of 5xFAD/CD33KO animals treated with K11. In cortex, CD33KO is mainly causative for the reduction in Aβ plaque counts. 

## 4. Discussion

Passive immunotherapy represents one of the most promising strategies for the treatment of AD. Although several attempts failed initially, e.g., with monoclonal antibodies Bapineuzumab or Crenezumab, there is a better understanding of the timepoint of initiation of treatment and thus a higher probability of success for antibodies currently under development. Several general mechanisms are proposed for how antibodies remove Aβ from the brain. Among those, microglial phagocytosis [29,30,31], the peripheral sink hypothesis [32,33,34] or catalytic disaggregation (“direct action hypothesis”), were anti-Aβ antibodies bind directly to senile plaques and destabilize their aggregates and eventually disrupt them [6]. Peripherally administered antibodies binding to non-modified Aβ peptides might act via a peripheral sink effect, because they first bind freshly synthesized and circulating Aβ peptides in the periphery [12,28]. The residual amount of these antibodies passing the BBB might be incapable of binding directly to amyloid plaques due to the fact that they are neutralized by plaque-surrounding Aβ molecules. In case of targeting modified Aβ peptides, microglial phagocytosis is likely to be the main mechanism of passive immunotherapy [12,35]. Since modifications such as isoaspartate formation mainly occur in the course of long-term storage of Aβ peptides [12], binding in the periphery is less likely. Consequently, more antibody molecules are available to enter the BBB. We could already show that our anti-isoD7-Aβ antibody K11 enters the BBB as it was detectable in the cerebellum 7 days after intraperitoneal treatment with 12 mg/kg. Above that, application of non-modified Aβ-specific antibody 3D6 leads to an accumulation of antibody–Aβ immune complexes in the periphery, resulting in lower antibody concentrations found in the cerebellum [12]. Once at the site of Aβ accumulation, antibodies binding post-translationally modified Aβ are less distracted by plaque-surrounding peptides, because just a small number of them are post-translationally modified. This enhances their probability to reach the deposited Aβ, thereby effectively inducing phagocytosis by brain-resident microglia and possibly infiltrating macrophages. Supporting this mode of action by using adequate drug combinations might help to overcome the drawbacks of Aβ-directed immunotherapy, e.g., by application of lower antibody doses. 

In order to assess one potential mechanism of enhanced Aβ clearance from brain, we here analyzed the effect of passive immunotherapy in 5xFAD/CD33KO mice. Since epidemiological studies suggested a protective effect of reduced expression of CD33 in human AD and previous studies in mice provided evidence for a role of microglia in that mechanisms, we here wanted to address whether a combination of CD33 reduction and passive immunotherapy exerts an additive effect on lowering Aβ deposition. 

In summary, our data support such a hypothesis, as the treatment of 5xFAD/CD33KO mice using the isoD7-Aβ antibody K11 showed an enhanced reduction in Aβ compared to 5xFAD mice treated with the antibody or 5xFAD mice harboring a CD33 null background. In more detail, the results presented in Figure 3 demonstrate a significantly enhanced number of plaque-surrounding microglia (*p* = 0.026 in cortex and *p* = 0.022 in hippocampus) when combining both treatments in comparison to the 5xFAD/CD33KO/Isotype group. There is also a visible tendency of enhanced plaque-surrounding microglia after K11 single therapy, but CD33KO alone even shows a slightly contrary effect within a 5xFAD background, pointing to a synergistic mode of action when combining both treatments. Interestingly, 5xFAD/CD33KO alone without any passive immunotherapy already shows an impact on isoD7-Aβ level in comparison to total Aβ level (Figure 1 and Figure 2). This indicates that plaques containing older Aβ with more post-translationally modified peptides are effectively removed by microglial phagocytosis, already without the need of passive immunotherapy. However, the large number of smaller, probably just emerging, plaques also accounts for the total Aβ pool, which is determined by ELISA and immunohistochemical analyses. This might contribute to an overall lower reduction in total Aβ compared to isoD7-modified Aβ. In conclusion, CD33KO alone influences phagocytosis of amyloid plaques in 5xFAD once microglia cells recognize sites of amyloid deposition, but the additional application of plaque-binding antibodies leads to the improved attraction of microglia to the plaque.

In addition to the increase in plaque-surrounding microglia by combinational treatment, CD33KO and isoD7-Aβ co-treatment significantly (*p* = 0.0174) reduces the number of Aβ plaques, but shows a marginal impact on plaque size (Figure 4). This again supports the assumption that microglial phagocytosis is the mode of action for K11 antibody treatment, rather than catalytic disaggregation. Once attracted to a plaque, microglia cells most likely phagocyte the whole plaque and not only parts of it. Furthermore, this theory is substantiated by the fact that the number of plaque-surrounding microglia rises significantly after combining K11 and CD33KO. 

If antibody K11 and CD33 act in the same pathway, CD33KO should promote the antibody-mediated effect in a synergistic manner. Providing evidence of the superiority of a combination of drugs compared to the single agents is a challenging field with a number of theoretical and experimental literature [36,37,38]. Two compounds act synergistically if their combination achieves greater effects than the simple additive effect expected from the knowledge of the effects of each drug individually [38]. However, definition of additivity is a tricky problem, mainly due to the fact that individual dose–effect curves are not linear. Indeed, most of them are characterized by logistic or curvilinear shapes [39]. In order to study drug combination effects, one has to be aware of the individual dose–effect curves of both agents, to define the concentration of each of them to produce the same quantitative impact [38,40]. Since we used CD33KO mice to study the impact of CD33 in the 5xFAD mouse model, we assume the individual drug maximum effect without knowing the dose–effect curve. 

CD33KO already mitigated Aβ plaque pathology in APPSwe/PS1ΔE9/CD33−/− mice [22], so we also expected attenuation of pathology in 5xFAD/CD33KO mice. For this reason, we decided to use a subtherapeutic dose for antibody-mediated isoD7-Aβ targeting. By some evaluation methods (please see Figure 2a–c and Figure S2b), a visible effect of the combinational therapy was demonstrated while treatment with minimal antibody dose alone shows no impact. Above that, the combination of antibody K11 and CD33KO shows in every case a higher effect than the maximum effect of CD33KO alone. Summarizing, we could clearly demonstrate an add-on effect, without being able to decide on a simple additive or a synergistic relationship between both approaches. An attempt to prove the synergistic action or additivity might be achieved by a combination of CD33 antibodies or inhibitors.

In this regard, a number of anti-CD33 antibodies/inhibitors are already available. Gemtuzumab ozogamicin, a humanized murine IgG4 anti-CD33 antibody, is the first antibody–drug conjugate approved for acute myeloid leukemia (AML) [41]. Another anti-CD33 antibody (Lintuzumab) was proven to be safe in humans [42]. Furthermore, an increasing number of CD33-directed Chimeric Antigen Receptor (CAR) T cells for the treatment of AML are currently tested in clinical trials [43]. In addition to antibody-based drugs, small molecules binding to the sialic acid ligand-binding region have been developed and shown to increase the uptake of Aβ into microglial cells [44]. Hence, it might be worthwhile to explore if the repurposing of anti-CD33 antibodies/inhibitors developed for treating AML is also effective for AD, especially in combination with antibodies targeting post-translationally modified Aβ.

The study presented here has some limitations which may be addressed in future studies, caused by the limited number of animals as well as the selection of one single treatment dose for every compound. The proof of synergistic action of both treatment strategies might be obtained in future studies by evaluating the single dose ratios which confirm and optimize the supposed synergy [45]. Although mouse CD33 is the apparent orthologue of human CD33, substantial species differences in CD33 expression patterns and ligand recognition have been reported [24]. This raises the question whether the influence of CD33 on AD-like pathology in mice can be translated to the influence of CD33 in the human body. Nevertheless, by examining the APP(Swe)/PS1(ΔE9)/CD33KO mouse line, a comparable role of murine CD33 in phagocytosis of amyloid plaques was already proven and again confirmed by our results [22]. 

## 5. Conclusions

Co-targeting CD33 when conducting passive immunization by using an antibody against post-translationally modified Aβ may have several advantages: (I) combinational therapy results in an add-on effect on passive immunotherapy alone; (II) anti-CD33 antibodies/inhibitors have already been developed for treatment of AML and might be easily repurposed for application in AD; (III) the success rate in clinical trials might be increased by the selection of patients carrying the AD-associated risk allele rs3865444C in the proximal promoter of CD33. (IV) Combinational therapy allows a reduction in anti-Aβ antibody dosage, thereby preventing side effects, since monoclonal antibodies showed ARIA-H and ARIA-E in clinical trials in a dosage-dependent manner.

## Data Availability

The data presented in this study are available on request from the corresponding authors.

## Supplementary Materials

The following supporting information can be downloaded at: https://www.mdpi.com/article/10.3390/biom12030399/s1, Figure S1: Quantification of total Aβ and isoD7-Aβ peptides in insoluble 5 M Guanidine hydrochloride (GdmCl) brain fractions of 5xFAD mice treated with different doses of K11_IgG2a.; Figure S2: Elevated Plus Maze (EPM) test of 5xFAD and 5xFAD/CD33KO mice treated weekly with 4 mg/kg K11_IgG2a or isotype control.

## Abbreviations

Acute myeloid leukemia: AML; Alzheimer’s disease: AD; amyloid beta: Aβ; amyloid precursor protein: APP; amyloid-related imaging abnormalities: ARIA; blood–brain barrier: BBB; CD33 knock out: CD33KO; Chimeric Antigen Receptor: CAR; Elevated Plus Maze test: EPM; familial AD: FAD; genome-wide association studies: GWAS; guanidine hydrochloride: GdmCl; heterozygous 5xFAD mice carrying a homozygous CD33KO: 5xFAD/CD33KO; L-Isoaspartate: isoD; pE: pyroglutamate; mild cognitive impairment: MCI; region of interest: ROI; standard error of the mean: SEM; 3,3′-diaminobenzidin: DAB.
