# Supplementary material for: Evidence for Enhanced Efficacy of Passive Immunotherapy against Beta-Amyloid in CD33-Negative 5xFAD Mice
Source: Biomolecules. 2022 Mar 4;12(3):399. doi: 10.3390/biom12030399 (PMC8945487; doi:10.3390/biom12030399)
Supplement: Supplementary file 1 [file biomolecules-12-00399-s001.zip › Supplementary Figures/proofed figure S1.pdf]

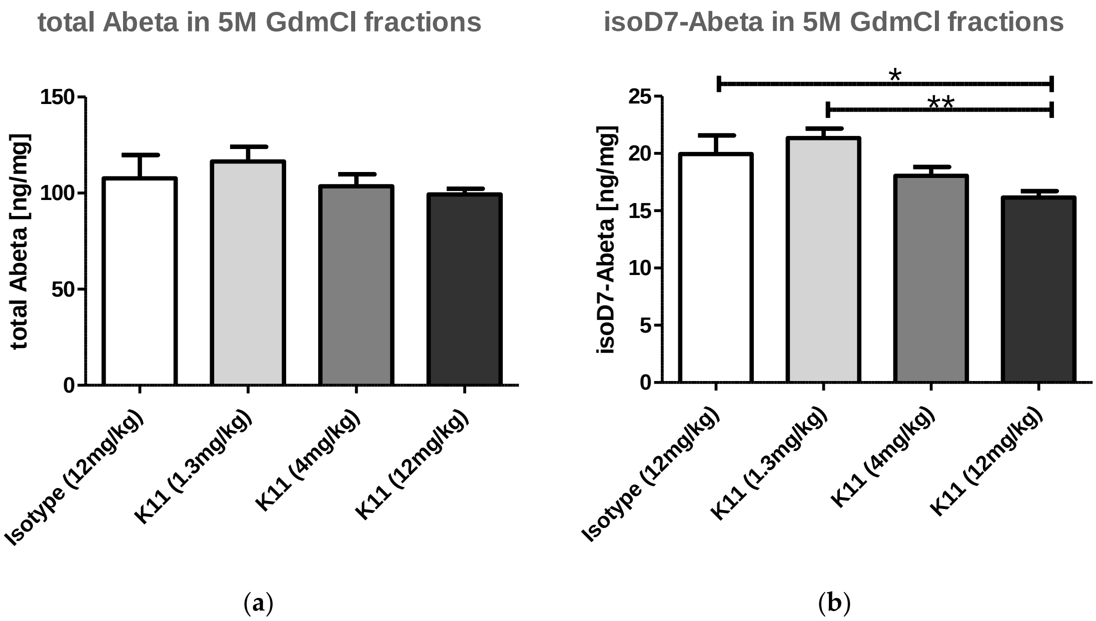

Figure S1 - Quantification of total Aβ and isoD7-Aβ peptides in insoluble 5 M Guanidine hydrochloride (GdmCl) brain fractions of 5xFAD mice treated with different doses of K11_IgG2a. Three-month-old 5xFAD mice were treated intraperitoneally once a week with 12, 4 or 1.3 mg/kg K11_IgG2a or 12 mg/kg isotype control, respectively. After 24 weeks of treatment, mice were sacrificed. The left hemisphere was homogenized in T-Per buffer, followed by centrifugation. The pellet was resuspended in 5 M GdmCl, again centrifuged and the supernatants applied to a total Aβ **(a)** and isoD7-Aβ **(b)** specific ELISA. Sample size was at least 8 animals per group. * means $P \leq 0.05$; ** means $P \leq 0.01$. The error bars represent SEM.
