# Supplementary material for: Evidence for Enhanced Efficacy of Passive Immunotherapy against Beta-Amyloid in CD33-Negative 5xFAD Mice
Source: Biomolecules. 2022 Mar 4;12(3):399. doi: 10.3390/biom12030399 (PMC8945487; doi:10.3390/biom12030399)
Supplement: Supplementary file 1 [file biomolecules-12-00399-s001.zip › Supplementary Figures/FigureS2.pdf]

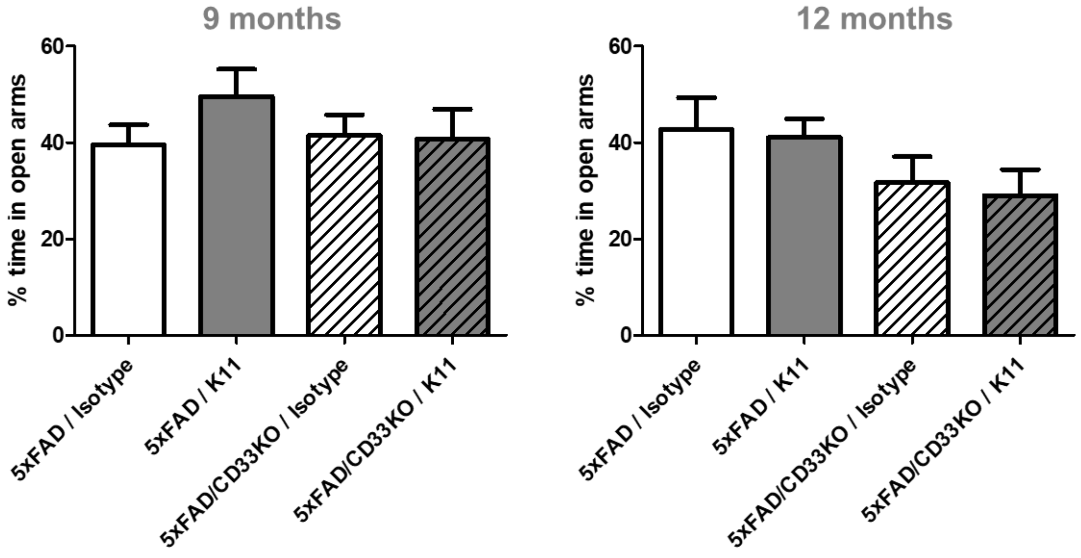

(**a**) (**b**)

Figure S2 - Elevated Plus Maze (EPM) test of 5xFAD and 5xFAD/CD33KO mice treated weekly with 4 mg/kg K11_IgG2a or isotype control. This test was performed 2 times with each animal, at the age of **(a)** 9 months and **(b)** 12 months. Test animals were placed with their head to the end of a defined closed arm of an elevated, plus-shaped (+) maze with two open and two enclosed arms. During the next 10 minutes, every movement of test animals was recorded by a video tracking system. The time the animals spent in the open arms was summed up in order to calculate % in exposed area. Sample size was at least 8 animals per group.
